# An instrument to measure nurses' knowledge in palliative care: Validation of the Spanish version of Palliative Care Quiz for Nurses

**DOI:** 10.1371/journal.pone.0177000

**Published:** 2017-05-18

**Authors:** Elena Chover-Sierra, Antonio Martínez-Sabater, Yolanda Raquel Lapeña-Moñux

**Affiliations:** 1 Nursing Department, University of Valencia, Valencia, Spain; 2 Hospital General Universitario de Valencia, Valencia, Spain; 3 Nursing Department, University Jaume I, Castellón de la Plana, Spain; Universite de Bretagne Occidentale, FRANCE

## Abstract

**Background:**

Palliative care is nowadays essential in nursing care, due to the increasing number of patients who require attention in final stages of their life.

Nurses need to acquire specific knowledge and abilities to provide quality palliative care. Palliative Care Quiz for Nurses is a questionnaire that evaluates their basic knowledge about palliative care.

The Palliative Care Quiz for Nurses (PCQN) is useful to evaluate basic knowledge about palliative care, but its adaptation into the Spanish language and the analysis of its effectiveness and utility for Spanish culture is lacking.

**Purpose:**

To report the adaptation into the Spanish language and the psychometric analysis of the Palliative Care Quiz for Nurses.

**Method:**

The Palliative Care Quiz for Nurses-Spanish Version (PCQN-SV) was obtained from a process including translation, back-translation, comparison with versions in other languages, revision by experts, and pilot study. Content validity and reliability of questionnaire were analyzed. Difficulty and discrimination indexes of each item were also calculated according to Item Response Theory (IRT).

**Findings:**

Adequate internal consistency was found (S-CVI = 0.83); Cronbach's alpha coefficient of 0.67 and KR-20 test result of 0,72 reflected the reliability of PCQN-SV. The questionnaire had a global difficulty index of 0,55, with six items which could be considered as difficult or very difficult, and five items with could be considered easy or very easy. The discrimination indexes of the 20 items, show us that eight items are good or very good while six items are bad to discriminate between good and bad respondents.

**Discussion:**

Although in shows internal consistency, reliability and difficulty indexes similar to those obtained by versions of PCQN in other languages, a reformulation of the items with lowest content validity or discrimination indexes and those showing difficulties with their comprehension is an aspect to take into account in order to improve the PCQN-SV.

**Conclusion:**

The PCQN-SV is a useful Spanish language instrument for measuring Spanish nurses’ knowledge in palliative care and it is adequate to establish international comparisons.

## Introduction

The WHO defined palliative care as the approach which improves the quality of life of patients and their families facing the problems associated with terminal illness through the prevention and relief of suffering by means of early identification and adequate assessment and treatment of pain and other physical, psychological and spiritual problems [1,2].

The recommendations given by the European Parliament in 2008 highlighted the importance of the development of specific palliative care plans in each country which should involve both professionals and citizens alike to guarantee the accessibility of services, promote comprehensive networks that ensure an efficient use of resources and health professionals training, within university curricula as well as ongoing professional training [3,4].

In 2007, the Spanish Ministry of Health published a strategy to develop palliative care in the Spanish Healthcare System. In this document they analyzed the current situation of palliative care and gave some indications for its development; this strategy was revised again in 2011, with recommendations to be undertaken in the period between 2011 and 2014, which included aspects such as patients who are susceptible to receive palliative care, material or human resources to provide quality palliative care as well as guidelines to evaluate palliative care plans and aspects regarding research in this field. Therefore, the Spanish regulations show the need to combine two types of strategies: basic formation of all health professionals in palliative care and the design of specific programs in this area to enable professionals to allow them the possibility of intervention at all assistance levels [4–6].

Nurses have always been considered a key element in providing palliative care throughout the life cycle; so an adequate training for nurses in palliative care and the involvement of the agencies responsible for providing this training is required.

The European Association for Palliative Care (EAPC), after analyzing nursing training programs available in various countries, formulated a guide for the development of nurses’ training in palliative care throughout Europe, with recommendations directed especially for those countries where palliative care was under development. They recommended the development of three levels of training: basic training for all nurses, intermediate qualification for nurses who care at times for patients requiring palliative care and specialized training for those working in services of palliative and end-of-life care [7–9]. In Spain, training in palliative care is included in degree programs of the majority of Nursing Schools, although in very different ways, reflecting the heterogeneity of this training [10]. There are also some Universities and agencies related to palliative care (as the Spanish Society of Palliative Care) that offer postgraduate and ongoing training with a variety in the hours of training offered and with contents related to the different levels of training. These data suggest us that the education around palliative care may be insufficient in some aspects and a screening is needed prior to elaborating strategies to improve knowledge in this important point in nurse education.

Thus, there is a need to analyze the level of knowledge in palliative care among Spanish nurses, and to measure this using a standardized and validated instrument that allows us to make comparisons not only between the various studies carried out in Spain, but also with other studies carried out in other environments.

### Background

As the development of a scale is a costly and lengthy process, nowadays it is a common practice to search for tools developed in different cultures to adapt and validate them in the new context where they are intended to be used [11]. Therefore, we decided to search for a validated document, which had been used to measure the level of knowledge of nurses in the field of palliative care even if it was in another language.

The Palliative Care Quiz for Nursing (PQCN), developed and validated by professors from the University of Ottawa, is a self-administered questionnaire that consists of 20 multiple-choice items (true/false/don’t know) and that assesses different aspects of palliative care [12].

The purpose of this study was to cross-culturally adapt the PCQN into the Spanish language and assess its psychometrics properties on a sample of Spanish registered nurses (RNs).

## Methods

### Design

A methodological design to develop the validity and reliability of the Spanish version of PCQN was used in this study.

Several authors have developed this methodology for cross-cultural adaptation of instruments, with slight modifications according to the purposes of their studies [11], [13–22]. We have used a methodology consisting of the following phases to obtain a version of the PCQN in Spanish:

#### Translation and back-translation

This two stages were performed by professional translators and Spanish RNs with advanced knowledge of English language, obtaining a consensus PCQN-Spanish version (PCQN-SV).

#### Experts review

Selected experts in the field of palliative care in Spain evaluated the adequacy and relevance of each item that composed the PCQN-SV on a scale of 1 (not suitable/not relevant) to 4 (very suitable/very relevant)

#### Content validity analysis

Content validity index of each item (I-CVI) and the global questionnaire CVI (S-CVI) were calculated on the basis of experts’ assessments; I-CVI were also used to stablish modified kappa coefficients (k).

#### Pilot study

A descriptive cross sectional study, developed in a Spanish tertiary hospital, designed to use the PCQN-SV for the first time, in order to identify problems in its comprehension and to analyze its psychometric properties and its internal consistency.

Participants in this pilot study were also asked to reflect any misunderstandings or difficulties found within the PCQN-SV.

The members of the experts group who revised the Spanish version of the PCQN were selected among nurses with a minimum of five years of experience in the field of palliative care, either working in the Spanish healthcare system or university professors teaching palliative care.

The population likely to participate in the pilot study was composed by those RNs who were on active duty during July 2014 in different units of a public hospital in Spain including the emergency department and critical care unit (a population of about 350 RNs). The objective was to collect approximately 160 questionnaires, through a purposive sampling. The sample size necessary for the study should had been calculated to be 153 subjects, with an error percentage of 5% and confidence interval of 90%.

### Data collection

- Evaluation by experts. Experts received an e-mail explaining the project and requesting their collaboration; on acceptance, the consensus PCQN-SV was sent by e-mail to them, with instructions to evaluate each item and to add their comments about its wording and its relevance to the field of palliative care.
- Pilot study. We used an own-developed document to collect two types of data.
  - Professional and sociodemographic data of participants (age, sex, level of training and years of experience as well as hours of training in the field of palliative care).
  - PCQN-SV. In addition to the responses to the questionnaire they could also identify any problems with items’ comprehension, providing their personal comments

The experts’ assessments were collected through questionnaires sent and received by email.

Nurses participating in the pilot study were notified of the purpose of the study with a letter attached to the questionnaire; the researchers informed them also through making periodic rounds on the units. The questionnaires used in this study were distributed in the various participating units: medical and surgical wards, emergency unit, intensive care unit, maternity and pediatrics. The researchers returned to the units on a regular basis to collect completed questionnaires, and an informative letter explaining the study’s purpose was attached to the questionnaires.

Data from experts were collected in the months of June and July 2014; whereas the month of January 2015 was devoted to the pilot study.

The pilot study was reviewed and approved by the Ethics and Research Committee of the hospital where it was conducted, CEIC-Hospital General Universitario, Valencia, on 18 December, 2014.

To preserve confidentiality and data anonymity, no personally identifying information was collected, and each document was identified with a numeric label to ease their handling.

Approval of the authors to adapt the original scale was also obtained, by contacting them via e-mail.

### PCQN

The Palliative Care Quiz for Nursing (PQCN) assesses three aspects of palliative care: philosophy and principles (4 items), control of pain and other symptoms (13 items) and psychosocial aspects (3 items). These 20 items refer to a knowledge that is applicable in clinical settings, and it could be submitted to both students and professional nurses [12].

According to various research studies, the different versions of PCQN have shown to be a useful instrument to measure nurses level of knowledge and also to identify misconceptions in the field of palliative care [23–33]. The studies to assess the validity of the various versions of PCQN in Korean, Dutch, French and Persian (Farsi) revealed appropriate internal consistency rates in any of the cases in which this issue was analyzed [29–32] [34]

This questionnaire has an internal consistency of 0.78 measured with KR-20 test (indicative of its homogeneity), measured in a heterogeneous group including student nurses, registered nurses and registered practical nurses with variations in their training about palliative care and years of experience in nursing [29]. It has also shown correlation coefficients higher than 0.5, in Pre-test/Post-test assessment designs developed in different contexts, considered by the authors as suitable coefficients [12,32]. In other versions of the PCQN they used Cronbach’s coefficient to assess its internal validity, obtaining values between 0,60 and 0,78, indicating also high internal validity.

### Translation of PCQN

**Translation**: The original version in English [12] was translated into the Spanish language by two independent translators and a Spanish RN with advanced knowledge of English, who worked in an oncology unit in a British Hospital. These three translations developed independently underwent a process of joint analysis by three translators to develop a unique version of the questionnaire on the basis of a consensus which was later revised by the researchers and other health professionals currently working in the field of palliative care.

**Comparison of translation with versions in other languages**: The consensus translation was compared with the French version of the PCQN to assess their equivalence, which also helped the research group to solve some translation difficulties.

**Back-translation**: The consensus Spanish version of PCQN (PCQN-SV) was back translated to the English language by two professional translators and a Spanish RN who has worked in an American hospital during the last five years. Thus, this back-translation process resulted in three new documents in English, from which a single document was agreed; this document was then compared item by item with the original PCQN by a different translator, to identify new discrepancies.

### Testing validity of PCQN-SV

Following the methodology described by Polit and Beck [35] and used by other authors [20–22] the content validity index of each of the items (I-CVI) was calculated individually, on the basis of the assessments carried out by the group of experts, using the following formula:

$$I-CVI=\frac{number\;of\;experts\;who\;evaluated\;the\;item\;with\;3\;or\;4}{Total\;of\;experts}$$

Then, the global questionnaire content validity index (S-CVI), defined as the arithmetic mean of the I-CVI was obtained. CVI values which are equal to or greater than 0.78 are considered acceptable, whereas values equal to or greater than 0.90 are considered indicative of high content validity [35]

Finally, the modified kappa coefficient (k), also considered an indicator of content validity, was calculated, to measure the level of agreement observed among experts; this coefficient, which obtains values between 0 and 1 is calculated with this formula:

$$\text{k}=\frac{\left(I-CVI\right)-Pc}{1-Pc}$$

In which the I-CVI is the coefficient of internal validity, previously calculated for each individual item, whereas the Pc (probability of chance agreement) is the probability of chance in the accordance between observers and is calculated through the formula:

$$Pc=\left[\frac{\left[N!\right]}{\left[A!\left(N-A\right)!\right]}\right]\times 0,5^{N}$$

The criteria to determine the level of agreement among the expert calculated with the kappa coefficient were established by Polit (kappa value ≥0, 74 excellent agreement; 0, 60≥ kappa value <0, 74 good agreement; kappa value <0, 59 poor agreement) [20,35].

### Testing reliability of PCQN-SV

This pilot study also enabled us to analyze the internal consistency of the questionnaire with the calculation of Cronbach’s alpha, and KR-20 test, to measure the reliability of the instrument;

A Cronbach’s alpha of 0.7 is set as the minimum acceptable value, and lower values indicate a low internal consistency of an instrument, although some authors consider that in the early stages of an investigation and in exploratory researches a value between 0.5 and 0.6 may be considered as suitable [36].

We performed also KR 20 test to assess the reliability of PCQN, as Dr Ross et al. did with the original PCQN, and with the French version, having into account that the PCQN can be considered as a dycotomic test (1 = right; 0 = wrong/don’t know).

### Testing difficulty of PCQN-SV

With the amount of wrong and right answers obtained by the subjects participating in pilot study and following the indications of the Item Response Theory (IRT), difficulty and discrimination indexes of each item composing the PCQN were calculated, using the top 33% and the bottom 33% of the results (in this case, we used the number of right answers given by the 50 best participants and the 50 worst ones), and according to these formula:

$$\text{Difficulty index}=\frac{\text{Right answers in best group}+\text{Right answers in worst group}}{\text{n best group}+\text{n worst group}}$$

$$\text{Discrimination index}=\frac{\text{Right answers in best group}-\text{Right answers in worst group}}{\text{n }\left(\text{best or worst group}\right)}$$

### Statistical analysis

CVI, Pc and kappa indexes were calculated using a database created in Excel 2013, using the evaluations from the Group of experts, and in accordance with their respective formula. Difficulty and discrimination indexes were also calculated with Excel 2013.

Other statistical analyses (including calculating Cronbach index and KR-20) were performed with the SPSS statistical package, version 20 (SPSS Inc., Chicago).

## Findings

### PCQN translation process

Clarification of items in the PCQN was required during the process of translation from English to Spanish; incidences are listed below.

Item 8. *Individuals who are taking opioids should also follow a bowel regime*. A query arose with the translation of the term bowel regime. Consultations with physicians in palliative care unit, resulted in the use of the broader expression “measures to improve the intestinal evacuation”.

Item 16. *Demerol is not an effective analgesic in the control of chronic pain*. Considering that Demerol is one of the trade names of meperidine, in the Spanish version Dolantina was used, because this drug is distributed in Spain under this name, and we consider that Spanish nurses would identify with it more easily.

Item 17. *The accumulation of losses renders burnout inevitable for those who seek work in palliative care*. It was understood that this statement referred to people already working in palliative care and this assumption was taken into account in the translation. In the French version of the questionnaire this item also referred to those working in palliative care.

### PCQN-SV

Table 1 shows the items composing the consensus PCQN-SV sent to the experts and also used in the pilot study, together with the original English PCQN.

**Table 1:** Spanish and English versions of PCQN.

1	Los cuidados paliativos son apropiados sólo en situaciones en las que hay evidencia de empeoramiento o deterioro de la situación clínica. *Palliative care is appropriate only in situations where there is evidence of a downhill trajectory or deterioration*
2	La morfina es el estándar utilizado para comparar el efecto analgésico de otros opioides *Morphine is the standard used to compare the analgesic effect of other opioids*
3	La extensión de la enfermedad determina el método de tratamiento del dolor. *The extent of the disease determines the method of pain treatment*
4	Las terapias adyuvantes son importantes en el manejo del dolor. *Adjuvant therapies are important in managing pain*
5	Es primordial para los miembros de la familia permanecer al lado del enfermo hasta su fallecimiento. *It is crucial for family members to remain at the bedside until death occurs*
6	Durante los últimos días de vida, la somnolencia asociada al desequilibrio electrolítico puede disminuir la necesidad de sedación. *During the last days of life*, *the drowsiness associated with electrolyte imbalance may decrease the need for sedation*
7	La adicción es un gran problema cuando se usa morfina como tratamiento de base para el manejo del dolor a largo plazo. *Drug addiction is a major problem when morphine is used on a long-term basis for the management of pain*
8	Los individuos que toman opioides deberían seguir medidas para mejorar la evacuación intestinal. *Individuals who are taking opioids should also follow a bowel regime*
9	Para proporcionar cuidados paliativos se necesita establecer un distanciamiento emocional. *The provision of palliative care requires emotional detachment*
10	Durante las fases finales de una enfermedad, los fármacos que pueden causar depresión respiratoria son apropiados para tratar la disnea severa. *During the terminal stages of an illness*, *drugs that can cause respiratory depression are appropriate for the treatment of severe dyspnea*
11	Los hombres, generalmente, resuelven su duelo más rápido que las mujeres. *Men generally reconcile their grief more quickly than women*
12	La filosofía de los cuidados paliativos es compatible con tratamientos activos. *The philosophy of palliative care is compatible with that of aggressive treatment*
13	El uso de placebos es apropiado en el tratamiento de algunos tipos de dolor. *The use of placebos is appropriate in the treatment of some types of pain*
14	A dosis altas, la codeína causa más náuseas y vómitos que la morfina. *In high doses*, *codeine causes more nausea and vomiting than morphine*
15	Sufrimiento y dolor físico son sinónimos. *Suffering and physical pain are synonymous*
16	La dolantina no es un analgésico efectivo en el control del dolor crónico. *Demerol is not an effective analgesic in the control of chronic pain*
17	La acumulación de pérdidas hace que el burnout sea inevitable para aquellos que trabajan en cuidados paliativos. *The accumulation of losses renders burnout inevitable for those who seek work in palliative care*
18	Las manifestaciones del dolor crónico son diferentes de las del dolor agudo. *Manifestations of chronic pain are different from those of acute pain*
19	La pérdida de alguien con quien se tiene una relación distante o conflictiva es más fácil de resolver que la de alguien con quien se tiene una relación más cercana o intima. *The loss of a distant or contentious relationship is easier to resolve than the loss of one that is close or intimate*
20	El umbral del dolor se ve reducido por la ansiedad o la fatiga. *The pain threshold is lowered by anxiety or fatigue*

### Experts review and content validity

We received 17 experts’ evaluations, nine of these experts developed their professional activity in the Spanish health system, while six of them combined their job in palliative care health units with part-time university teaching and only two of them were full-time professors, all within the field of palliative care.

The values of CVI, and Kappa for each of the items composing the questionnaire, calculated from their 17 evaluations, are shown in Table 2.

**Table 2:** CVI and kappa index calculated for each item.

Item	CVI	k
**1**	0,94	0,94
**2**	0,70	0,69
**3**	0,82	0,82
**4**	0,94	0,94
**5**	0,88	0,88
**6**	0,76	0,76
**7**	0,82	0,82
**8**	0,82	0,82
**9**	0,70	0,69
**10**	0,76	0,76
**11**	0,70	0,69
**12**	0,82	0,82
**13**	0,94	0,94
**14**	0,76	0,76
**15**	0,88	0,88
**16**	0,76	0,76
**17**	0,76	0,76
**18**	0,94	0,94
**19**	0,88	0,88
**20**	0,94	0,94

Analyzing the CVI of each item we see that 40% of them do not reach the value of 0,78 considered as acceptable; but among these, five of them have a CVI of 0.76, a little lower than 0.78.

If we calculate the global CVI of the questionnaire (S-CVI) we see that it has a value of 0.83, greater than the value defined as acceptable.

Regarding the kappa index, we can appreciate that most of the items (85%) have an excellent level of concordance, while the rest have good concordance levels.

Thus, we could say that PCQN-SV presents a suitable content validity, even though the items with the lowest indexes should be improved in order to increase its content validity.

### Internal validity

From the answers given by the participants in the pilot study, the percentage of right and wrong answers to each of the questions compiled within the questionnaire was analyzed, and then the Cronbach index was calculated, and KR-20 test was performed obtaining a result in KR-20 of 0,72 and a value of 0,67 for Cronbach index; a value lower than that considered to be adequate, but suitable for the use in the initial phases of the questionnaire [36].

Whilst analyzing the correlation between the elements, we can see that the Cronbach index would rise to 0,70 if we eliminated items 4, 8 and 10; however, we do not consider it necessary to remove them as these three items ask about pharmacological treatment and participants obtained good results in them.

### Pilot study results

A sample of 159 registered nurses throughout the study hospital completed the PCQN.

This was a group of professionals with an average age of 39 years and fourteen years of professional experience. Just over half reported having experience in palliative care and a higher percentage refer to have training in this area, although one third of them had received this training only during their university education and they have not received any other extra training.

Table 3 shows the characteristics of the studied population, socio-demographic data and information concerning their training and experience in palliative care.

**Table 3:** Demographic and professional characteristics of participants (n = 159)*.

Characteristics of participants	Mean ± SD	N	(%)
**Age**	39,51 ± 10,25		
**Sex**			
** Women**		134	84,28
** Men**		25	15,72
**Years of professional experience**	13.96 ± 10,79		
**Experience in palliative care**			
** YES**		87	54,7
** NO**		72	45,3
**Experience in palliative care (years)**	4,05 ± 4,74		
**Training in palliative care**			
** YES**		102	64,2
** NO**		57	35,8
**Training in palliative care (Type) (n = 102)**			
** University education**		32	31,4
** Ongoing training**		30	29,4
** University+Ongoing training**		24	23,5
** Postgraduate courses**		6	5,9
** Others**		5	4,9
**Training in palliative care (Hours)**			
** <20**		30	29,4
** 20–50**		33	32,4
** 50–100**		28	27,5
**>100**		11	10,8

*Data shown in this table have been previously published and are also available at Chover-Sierra E, Martínez-Sabater A, Lapeña-Moñux Y. Conocimientos en cuidados paliativos de los profesionales de enfermería de un hospital español. Rev Lat Am Enfermagem. 2017;25. (in press)

We calculated the percentage of right and wrong answers obtained by each participant; they obtained an average of 54%, of right answers (54,0 ± 1.1; Range 20–95) and a 33% of wrong answers (33.1 ± 0.9; Range 0–60). This would average 10.8 points from a maximum of 20 on the global questionnaire score.

The percentage of right and wrong answers was also calculated for every one of the items composing the questionnaire, resulting in a high variability among them. Items with higher percentage of right answers (over 80%) were items 1, 4, 8 and 18, and those with highest percentage of errors were ítems 5, 17 and 19 (more than 60% in the case of items 17 and 19 and higher than 80% for item 5).

The percentages of right and wrong answers and the difficulty and discrimination index for each item, calculated on the basis of the amount of right and wrong answers are shown in Table 4.

**Table 4:** Percentages of right and wrong answers and difficulty and discrimination indexes of each item.

			IRT	
Item	Right answers	Wrong answers	Difficulty	Discrimination
1	133 (83,6%)	25 (15,7%)	0,82	0,24
2	81 (50,9%)	36 (22,6%)	0,49	0,22
3	89 (56%)	60 (37,7%)	0,56	0,72
4	153 (96,2%)	3 (1,9%)	0,97	0,06
5	22 (13,8%)	133 (83,6%)	0,19	0,1
6	49 (30,8%)	70 (44%)	0,32	0
7	90 (56,6%)	64 (40,3%)	0,53	0,62
8	131 (82,4%)	12 (7,5%)	0,77	0,38
9	88 (55,3%)	60 (37,7%)	0,57	0,26
10	101 (63,5%)	41 (25,8%)	0,65	0,22
11	84 (52,8%)	26 (16,4%)	0,49	0,42
12	83 (52,2%)	51 (32,1%)	0,57	0,02
13	78 (49,1%)	66 (41,5%)	0,53	0,38
14	49 (30,8%)	22 (13,8%)	0,31	0,34
15	110 (69,2%)	41 (25,8%)	0,69	0,38
16	80 (50,3%)	48 (30,2%)	0,5	0,28
17	48 (30,2%)	96 (60,4%)	0,31	0,5
18	133 (83,6%)	23 (14,5%)	0,84	0,04
19	44 (27,7%)	104 (65,4%)	0,29	0,26
20	77 (48,4%)	71 (44,7%)	0,59	0,34

The analysis of the difficulty index shows that items 10, 15, 8, 1, 18 and 4 can be considered as easy (difficulty index from 0,61 to 0,80) or very easy (over 0,80), whilst the items 6, 17, 14, 19 and 5 are difficult (difficulty index from 0,21 to 0,40) or very difficult, and we can also affirm that the average difficulty of the PCQN-SV is 0,55.

Calculating the discrimination indexes help the researchers to find out which items could be defined as very good (indexes higher than 0,40; items 3, 7 and 11), good (indexes between 0,3 and 0,39; items 8, 13, 15, 14 and 40), fair (between 0,2 and 0,29; items 16, 9, 29, 1, 2 and 10) or bad (lower or negative discrimination indexes), and therefore rewriting this last group should be taken into consideration.

All of the results of the pilot study are shown and discussed in depth by the authors in a different paper, where they analyze the relationship between the results obtained in the PCQN and some characteristics of the study population, finding a significant association between the percentage of right answers and wrong answers and nurses’ experience and training in the field of palliative care with Pearson correlation test [37].

## Discussion

The authors needed a validated instrument to measure Spanish nurses’ knowledge in the field of palliative care, so they decided to develop and analyze validity and reliability of the Spanish version of a well-known instrument, the PCQN. The reasons to use the PCQN Spanish version as a tool to measure nurses’ knowledge in palliative care were: its shortness, its self-administration, the fact that it considers different aspects of palliative care and that it has been translated into several languages and that their different versions have shown appropriate internal validity levels, which has been useful in order to measure nurses level of knowledge about palliative care in different countries [23,25], [27–29], [31–34].

Regarding the content validity of PCQN-SV, we observed that up to eight items have a CVI below desirable values, although for five of them it was very close to the established limits. A reformulation of these items could be an aspect to take into account in order to improve its content validity, although it should not be forgotten that this questionnaire presents a global CVI considered as acceptable according to the criteria stablished in scientific literature.

In our case, the global CVI is 0.83, very similar to that obtained for the Korean version, which was 0.85 [29]. With regards to the Iranian version [34] we only know that ten experts evaluated it and considered it as adequate, but it is not indicated neither if the CVI has been calculated nor its value. Neither the CVI results of the French version [25] are known, although a group of experts evaluated it and the lowest valued items were revised.

To study the internal consistency of PCQN-SV both Cronbach and KR-20 indexes were calculated. The original version of PCQN presents adequate internal consistency levels through the KR-20; whilst this PCQN-SV has obtained a lower value than the original one, but higher than the French version with a KR-20 value of 0,60.

The Korean version [29], had a Cronbach index of 0,65, very similar to our results and also inferior to the original questionnaire of 0,78, and the French version [25] got a Cronbach index of 0,60. On the other hand, the Iranian (Farsi) version used in a study in three hospitals, also showed a Cronbach index of 0.78 [34]. It must not be forgotten that the analysis of reliability coefficient should be performed for each population where the questionnaire is used, and that these populations may not always be comparable with regard to their defining features [38].

With regards to the difficulty of the items, we could say that there is a balanced distribution of items in questionnaire (25% of difficult-very difficult, 25% of easy-very easy and 50% of medium difficulty) we were also able to compare our results with those of the original PCQN as well as with those of the French version of the PCQN [12,25]. Our questionnaire has an average difficulty index of 0,55, lower to the difficulty coefficients of the original English version (with an average difficulty coefficient of 0,75), but very similar to the French version (with an average difficulty coefficient of 0,56). Only six items of our questionnaire (1, 2, 3, 6, 12 and 18) have difficulty indexes higher (are easier) as their equivalents in the original version, while nine of them (1, 4, 7, 12, 14, 15, 16, 18, 20) have difficulty indexes higher as the French version.

Regarding the coefficient of discrimination, Ross et al., while explain the process of elaboration of PCQN talk about this index, however no coefficients are presented for each item; they explain that the only criteria to consider that an item is adequate to form part of the PCQN is its difficulty index [12]. In our case we found that six items had very low discrimination coefficients, therefore these should be eliminated from the questionnaire, however in the end they were not eliminated, so as to establish comparisons with other versions, and because these coefficients can also vary depending on the characteristics of those who answer the questionnaire, so it could be useful to understand which items discriminate the most between experts and non-experts within the area of palliative care, with the analysis of discrimination indexes in different populations.

In PCQN Dutch’s version [24] item 16 was eliminated due to its reference to the drug called ‘Demerol’, not used in their context; and in a study developed in Canada [27] with nurses working at residential aged care facilities, they substituted Demerol with morphine, considering in both assessments these versions as equivalent to the original.

From the results of the current study, a reformulation of item 5 has been proposed for use in further occasions, which would be written as ‘essential for all families’ or ‘essential for any family’, because with the actual translation there is a tendency for Spanish nurses to mark this assessment as true, which is an error. In the Korean version there was also a great percentage of errors in this item, reminding us about cultural barriers in understanding this assessment, which talks about the role of grieving family members. It is very difficult for Spanish nurses, influenced by their social-cultural reality, to answer this question in a professional and non-sentimental manner.

The results presented suggest that the PCQN-SV is a valid instrument to measure basic level of knowledge in the nursing profession in the field of palliative care in a quick and efficient way, due to its brevity and its capability for self-administration. However, revisions to some statements of this Spanish version are suggested to improve this tool to keep it in line with ongoing knowledge and clinical practice.

It would be interesting to assess whether the proposed changes in the wording of the questionnaire items could affect its validity, taking into account that the objective of these changes is to facilitate the comparison of results with those obtained in countries where other versions of PCQN were used.

### Limitations

Being our principal objective to analyze the validity and reliability of PCQN-SV, we only accessed in a limited population (nurses working in one Spanish hospital), therefore there is a need for further study with the performance of multicenter studies where larger populations with different characteristics can be observed, allowing analysis of reliability of the questionnaire in the different contexts in which it is evaluated.

It’s also a limitation don’t having perform a test-retest analysis in order to obtain more information about the reliability of PCQN-SV, a test that was performed in the validation of the original PCQN.

## Conclusion

The findings of this study support the reliability and validity of the PCQN-SV for its use among Spanish RNs to measure their basic knowledge in the field of palliative care. Although some modifications are needed in order to improve its psychometric properties, it could be useful to identify nurses’ basic knowledge of palliative care in different clinical and educational settings, and also which educational methods are associated with increased levels of knowledge.

It would also benefit the evaluation of public policies and educational programs aimed towards professionals and organized by health or educational organizations. Finally, the availability of the Spanish version of the PCQN enables international comparisons, studying the differential factors that act as determinants between countries and as the base for the validation of the questionnaire in other Spanish-speaking countries making appropriate linguistic adjustments if necessary.
