# Correction: An instrument to measure nurses' knowledge in palliative care: Validation of the Spanish version of Palliative Care Quiz for Nurses

**DOI:** 10.1371/journal.pone.0180622

**Published:** 2017-06-27

**Authors:** Elena Chover-Sierra, Antonio Martínez-Sabater, Yolanda Raquel Lapeña-Moñux

The affiliation for the second author is incorrect. Antonio Martínez-Sabater is not affiliated with #2 but with #1 Nursing Department, University of Valencia, Valencia, Spain.
